# MicroRNA-101 Modulates Autophagy and Oligodendroglial Alpha-Synuclein Accumulation in Multiple System Atrophy

**DOI:** 10.3389/fnmol.2017.00329

**Published:** 2017-10-17

**Authors:** Elvira Valera, Brian Spencer, Jennifer Mott, Margarita Trejo, Anthony Adame, Michael Mante, Edward Rockenstein, Juan C. Troncoso, Thomas G. Beach, Eliezer Masliah, Paula Desplats

**Affiliations:** ^1^Department of Neurosciences, University of California, San Diego, La Jolla, CA, United States; ^2^Department of Pathology, The Johns Hopkins University School of Medicine, Baltimore, MD, United States; ^3^Banner Sun Health Research Institute, Sun City, AZ, United States; ^4^Department of Pathology, University of California, San Diego, La Jolla, CA, United States

**Keywords:** microRNA, multiple system atrophy, autophagy, alpha-synuclein, miR-101

## Abstract

Synucleinopathies, neurodegenerative disorders with alpha-synuclein (α-syn) accumulation, are the second leading cause of neurodegeneration in the elderly, however no effective disease-modifying alternatives exist for these diseases. Multiple system atrophy (MSA) is a fatal synucleinopathy characterized by the accumulation of toxic aggregates of α-syn within oligodendroglial cells, leading to demyelination and neurodegeneration, and the reduction of this accumulation might halt the fast progression of MSA. In this sense, the involvement of microRNAs (miRNAs) in synucleinopathies is yet poorly understood, and the potential of manipulating miRNA levels as a therapeutic tool is underexplored. In this study, we analyzed the levels of miRNAs that regulate the expression of autophagy genes in MSA cases, and investigated the mechanistic correlates of miRNA dysregulation in *in vitro* models of synucleinopathy. We found that microRNA-101 (miR-101) was significantly increased in the striatum of MSA patients, together with a reduction in the expression of its predicted target gene *RAB5A*. Overexpression of miR-101 in oligodendroglial cell cultures resulted in a significant increase in α-syn accumulation, along with autophagy deficits. Opposite results were observed upon expression of an antisense construct targeting miR-101. Stereotaxic delivery of a lentiviral construct expressing anti-miR-101 into the striatum of the MBP-α-syn transgenic (tg) mouse model of MSA resulted in reduced oligodendroglial α-syn accumulation and improved autophagy. These results suggest that miRNA dysregulation contributes to MSA pathology, with miR-101 alterations potentially mediating autophagy impairments. Therefore, therapies targeting miR-101 may represent promising approaches for MSA and related neuropathologies with autophagy dysfunction.

## Introduction

Synucleinopathies are a group of neurodegenerative disorders characterized by the pathological accumulation of the synaptic protein alpha-synuclein (α-syn). Synucleinopathies include Parkinson’s disease (PD), PD dementia, dementia with Lewy bodies (DLB) and multiple system atrophy (MSA) and currently affect 1.5 million people in the United States alone, and to date there are not disease-modifying therapies available for these disorders. α-syn accumulates within neurons and glial cells in synucleinopathies, leading to neurodegeneration and neuroinflammation, and in some cases loss of trophic support (Spencer et al., 2016). Moreover, α-syn can propagate from cell to cell in a prion-like fashion thus spreading the pathology through the brain (Desplats et al., 2009; Lee et al., 2010; Olanow and Brundin, 2013; Masuda-Suzukake et al., 2014; Prusiner et al., 2015).

Considerable effort is being devoted to understanding the pathogenesis of PD, however less is known about less frequent synucleinopathies such as multiple system atrophy (MSA). MSA is a rapidly progressive and fatal neurodegenerative disease characterized by parkinsonism, dysautonomia (Dickson et al., 1999a; Wenning et al., 2001), and α-syn accumulation within oligodendroglial and neuronal cells (Lantos and Papp, 1994; Jellinger, 2012). This abnormal protein accumulation is accompanied by neuroinflammation (Stefanova et al., 2007; Valera et al., 2014), demyelination (Matsuo et al., 1998; Wenning et al., 2008) and neurodegeneration (Jellinger, 2003; Ubhi et al., 2011). While 80% of MSA patients present prominent parkinsonian features reflecting striato-nigral neurodegeneration (MSA-P subtype), the other 20% present cerebellar ataxia as a consequence of olivo-pontocerebellar atrophy (MSA-C subtype; Gilman et al., 2008). In both cases the rapid progression, lack of response to levodopa (Wenning et al., 1994), and extensive accumulation of α-syn within oligodendrocytes differentiate clinically and pathologically MSA from other synucleinopathies (Dickson et al., 1999b).

Importantly, the source of oligodendroglial α-syn in MSA is still unclear, and the molecular mechanisms favoring a greater accumulation of α-syn in oligodendrocytes than in neurons have not been identified. Given the high levels and widespread distribution of α-syn aggregates in MSA, it is possible that both propagation from other cell types (Kisos et al., 2012; Reyes et al., 2014; Valera et al., 2014) and oligodendroglial α-syn expression (Asi et al., 2014) might be occurring simultaneously. Regardless of the origin of α-syn, recent evidence supports the notion that failure of intracellular protein clearance mechanisms (e.g., autophagy, unfolded protein response, proteolysis) might play a role in the process of α-syn aggregation (Klucken et al., 2012; Lee et al., 2013), release to the extracellular environment (Lee et al., 2013), and accumulation in both donor and acceptor cells. Supporting this hypothesis, impairments in clearance mechanisms such as autophagy have already been described in MSA and other synucleinopathies (Lynch-Day et al., 2012; Schwarz et al., 2012). Autophagy is the main clearance mechanism for abnormal protein aggregates and organelles in the central nervous system (CNS; Wong and Cuervo, 2010), and it is affected in most diseases that show toxic accumulation of proteins, such as Alzheimer’s disease (AD; Pickford et al., 2008), PD (Cuervo et al., 2004; Crews et al., 2010) and DLB (Crews et al., 2010). It is not clear if autophagy dysfunction is the cause or a consequence of α-syn accumulation within those cells (Winslow and Rubinsztein, 2011). However, activation of autophagy has been shown to reduce α-syn accumulation and to improve behavioral deficits in animal models of the disease (Spencer et al., 2009; Crews et al., 2010).

The autophagy machinery can be regulated by transcriptional and epigenetic mechanisms, as well as post-transcriptional modifications such as phosphorylation and acetylation (Fullgrabe et al., 2014). In this context, microRNAs (miRNAs) have been identified as essential for a variety of cellular events including autophagy (Frankel and Lund, 2012; Fu et al., 2012). Autophagy-regulating miRNAs have been identified in cancer models, and they include miR-101, miR-30a, miR-34c and miR-183, among others (Zhu et al., 2009; Frankel et al., 2011; Frankel and Lund, 2012; Fu et al., 2012). Some of the predicted targets for miRNA regulation of autophagy are ULK1, mTOR, Beclin 1, LC3 and Atg proteins (Frankel and Lund, 2012; Fu et al., 2012). In PD, it has been proposed that the expression of LAMP-2A and Hsc70 (chaperone-mediated autophagy) is regulated by a subset of eight miRNAs (hsa-miR-21*, hsa-miR-379, hsa-miR-373*, hsa-miR-320a, hsa-miR-224, hsa-miR-301b, hsa-miR-26b and hsa-miR-106a; Alvarez-Erviti et al., 2013), however the mechanistic link between the dysregulation of miRNAs in synucleinopathies, autophagy deficits and α-syn propagation and accumulation in oligodendrocytes has not been studied.

Alterations in miRNA regulation have been implicated in the pathogenesis of neurodegenerative disorders. For example, up-regulation of specific miRNAs has been found in AD (Banzhaf-Strathmann et al., 2014; Tan et al., 2014), PD (Khoo et al., 2012; Vallelunga et al., 2014), frontotemporal dementia (Gascon and Gao, 2014), and MSA (Ubhi et al., 2014; Vallelunga et al., 2014), among others. Here we propose an *additional* mechanism regulating autophagy in MSA, involving miRNA-induced gene silencing. In this scenario, the dysregulation of miRNAs such as miR-101 that inhibit the expression of autophagy proteins would further inhibit the proper clearance of α-syn in oligodendrocytes. We found that miR-101 levels were significantly elevated in the striatum of MSA patients, and that miR-101 manipulation *in vitro* and *in vivo* was sufficient to effectively regulate autophagy and α-syn accumulation in oligodendrocytes. We conclude that therapeutic interventions targeting miR-101 and/or other autophagy-regulating miRNAs might be of use for the treatment of MSA and related disorders with autophagy dysfunction.

## Materials and Methods

### Human Brain Samples

Brain tissue samples were obtained from three institutions: University of California, San Diego Shiley-Marcos AD Research Center (UCSD-ADRC; *n* = 6); Johns Hopkins Medical Institution Brain Resource Center (*n* = 10); and Banner Sun Health Research Institute (*n* = 8; Birdsill et al., 2011; Beach et al., 2015). Samples included frozen tissue from the striatum, frontal cortex and cerebellum of controls (*n* = 7) and MSA patients (*n* = 17). The materials were collected and utilized with the written consent of the subjects, and the study conformed to The Code of Ethics of the World Medical Association (Declaration of Helsinki), printed in the British Medical Journal (Rickham, 1964). The parent study was reviewed and approved by the University of California, San Diego Human Research Protections Program and the corresponding Institutional Review Boards that oversee research at the collaborating brain banks. Animals were handled in strict accordance with the NIH guide for the care and use of laboratory animals (NIH publications no. 8023, revised 1978), and all procedures were completed under the specifications set forth by the UCSD Institutional Animal Care and Use Committee. Case selection for this study was based on neuropathological examination and determination of diagnosis of MSA-P (Gilman et al., 2008), which is the most common MSA variant in the Western hemisphere (Jellinger, 2014). Group demographics are presented in Supplementary Table S1.

### Lentiviral Vectors Expressing miR-101 and antimiR-101

Lentiviral vectors expressing rodent miR-101a or antimiR-101a, and their corresponding control vectors, were obtained from Genecopoeia. The vectors include a fluorescent reporter to monitor construct expression. Lentiviruses (LV) expressing miR-101 (rno-miR-101a-3p, mmu-miR-101a-3p, sequence UACAGUACUGUGAUAACUGAA), antimiR-101 (antisense sequence against miR-101a-3p), or their appropriate vector controls (miR-control or antimiR-control) were prepared by transient transfection in 293T cells (Tiscornia et al., 2006).

### Cell Culture

CG-4 is a bipotential cell line capable of differentiating into either oligodendrocytes or type 2-astrocytes, which in its undifferentiated state expresses oligodendroglial precursor markers such as A2B5, PDGFRα and Olig2 (Louis et al., 1992, and unpublished data). CG-4 cells were cultured as previously described (Louis et al., 1992). Briefly, cells were grown in serum free culture medium consisting in 70% DMEM containing 10% N1 supplement (Sigma) and 10 ng/ml biotin (Sigma), and 30% conditioned DMEM media from B104 cells (Schubert et al., 1974). Cells were grown in plates precoated with 0.1 mg/ml poly-L-ornithine. CG-4 cells were co-infected with LV expressing human α-syn (LV-α-syn) or LV control, and miR-101a-3p (LV-miR-101) or control vector (LV-miR-control; Bar-On et al., 2008) using a MOI ratio of 50, and were analyzed after 3 days of expression. For immunocytochemistry experiments, cells were plated onto poly-L-ornithine-coated glass coverslips at a cell density of 2.8 × 10^4^ cells per cm^2^ and fixed in 4% paraformaldehyde. For RNA extraction, protein extraction and immunoblotting, cells were plated onto 12-well plates at a cell density of 1.15 × 10^5^ cells per cm^2^.

### Animal Model and Stereotaxic Injections

Mice expressing human α-syn under the control of the myelin basic protein (MBP) promoter (MBP-α-syn) was generated as previously described (Shults et al., 2005). For the miRNA analysis of non-tg and MBP-α-syn tg mice, Line 29 (*n* = 5 per group), brains were sub-dissected into striatum, frontal cortex and cerebellum, snap-frozen in liquid nitrogen, and stored at −80°C for subsequent protein and RNA analysis.

For stereotaxic injections we used the MBP-α-syn Line 1, as they express an intermediate level of α-syn expression compared to Line 29, are more viable, less aggressive and tolerate surgery (Shults et al., 2005). Mice were injected bilaterally with 2 μl per injection site of LV-antimiR-101a or LV-antimiR-control (2.5 × 10^7^ TU) into the striatum (*n* = 6 each non-tg and MBP-α-syn tg). Briefly, as previously described (Ubhi et al., 2009), mice were placed under anesthesia on a Koft stereotaxic apparatus and coordinates (AP = +1 mm; L = ±1.5 mm, DV = −3.0 mm) were determined as per the Franklin and Paxinos Atlas. The lentiviral preparations were delivered using a Hamilton syringe connected to a Nano-injector system to inject the solution at a rate of 0.5 μl/min. To allow diffusion of the solution into the brain tissue, the needle was left on for an additional 5 min after the completion of the injection. Mice were 5 months at the time of the injections, and were sacrificed 6 weeks after injection under anesthesia, following NIH guide for the care and use of laboratory animals (NIH publications no. 8023, revised 1978). Brains were fixed by immersion in 4% paraformaldehyde in PBS pH 7.4 and sagittally sectioned at 40 μm with a Vibratome apparatus (Leica) for subsequent immunohistochemical analysis.

### Immunohistochemistry, Immunocytochemistry and Electron Microscopy

Vibratome sections or coverslips were immunolabeled overnight with an antibody against α-syn (recognizing both human and murine α-syn; Millipore, 1:200), followed by incubation with species-appropriate secondary antibody (Vector Laboratories). Sections or coverslips were reacted with 3,3′-diaminobenzidine (Vector Laboratories) and imaged on an Olympus BX41 bright field digital microscope. A minimum of 100 cells were counted per condition, and cell counts are expressed as the average number of positive cells per field (230 μm × 184 μm).

For autophagy analysis, sections were immunolabeled overnight with an antibody against LC3 (MBL International, 1:2500), followed by incubation with the species-appropriate secondary antibody and detection with the Tyramide Signal Amplification™-Direct (Red) system (1:100; Perkin Elmer). Sections were transferred to SuperFrost slides (Fisher Scientific) and mounted under glass coverslips with anti-fading media (Invitrogen) before analysis with a MRC1024 laser scanning confocal microscope (BioRad). Quantification of LC3 staining was performed by obtaining optical density measurements using the Image Quant 1.43 program (NIH) and corrected against background signal levels.

For electron microscopy, vibratome sections were post-fixed in 1% glutaraldehyde, treated with osmium tetraoxide, embedded in epon araldite and sectioned with the ultramicrotome (Leica). Grids were analyzed with a Zeiss OM 10 electron microscope as previously described (Masliah et al., 2011). Cells were randomly acquired from three grids, and electron micrographs were obtained at a magnification of 25,000×.

### Immunoblotting

Protein homogenates were prepared from frozen tissue samples and from cells grown in 12-wells plates. Briefly, whole protein samples were sonicated in homogenization buffer (HEPES 1 mM, benzamidine 5 mM, 2-mercaptoethanol 2 mM, EDTA 3 mM, MgSO_4_ 0.5 mM, NaN_3_ 0.05%, protease inhibitor cocktail set III 1:100, phosphatase inhibitor cocktail set II 1:100). Twenty microgram of protein were loaded onto 4%–12% Bis-Tris SDS-PAGE gels (Invitrogen), transferred onto Immobilon membranes. After overnight incubation with antibodies against α-syn (recognizing both human and murine α-syn; Millipore, 1:200), Beclin 1 (Novus Biologicals), LC3 (MBL International), or p62 (Cell Signaling), membranes were incubated in HRP-linked secondary antibody (American Qualex), reacted with ECL Western blotting substrate (Perkin Elmer) and developed in a VersaDoc gel-imaging machine (BioRad). Immunoblotting images were analyzed using Quantity One software (BioRad).

### Real Time PCR

Total RNA was extracted from the mouse anterior hemibrain using a Qiagen miRNeasy kit and following the instructions of the manufacturer. For quantitative real time PCR (qPCR), 0.5 μg of total RNA per sample were used for reverse transcription to cDNA using a High capacity cDNA reverse transcription kit (Applied Biosystems). cDNA was diluted 1:10 in ultrapure water and 4 μl of this dilution were used per reaction. qPCR was performed using TaqMan Fast Advanced Master Mix and species-specific TaqMan primers. The expression of the gene *ACTB* (beta actin) was used as internal control (Desplats et al., 2012). For miRNA qPCR, 10 ng of total RNA per sample were used for reverse transcription to cDNA using TaqMan MicroRNA Reverse Transcription Kit and miRNA-specific primers (Applied Biosystems), and 3 μl of cDNA were used per reaction. MicroRNA qPCR was performed using TaqMan Universal Master Mix II, no UNG and species-specific TaqMan miRNA primers, using U6 as internal control (Peltier and Latham, 2008). qPCR reactions were run in an StepOnePlus Real-Time PCR system (Applied Biosystems) and ∆∆Ct calculations were made using StepOne software (Applied Biosystems).

### Statistical Analysis

Differences between groups (*n* = 5–7) were tested using Student’s *t*-test, or one-way analysis of variance (ANOVA) with Tukey’s *post hoc* test. Linear correlation between two variables was measured using the Pearson correlation coefficient (Pearson’s *r*). For *in vitro* assays, all conditions were assayed in duplicate and repeated in at least three separated experiments. All results are expressed as average ± SEM.

## Results

### Levels of miRNAs that Reportedly Modulate the Expression of Autophagy Genes Are Dysregulated in the MSA Brain

**Figure 1:**
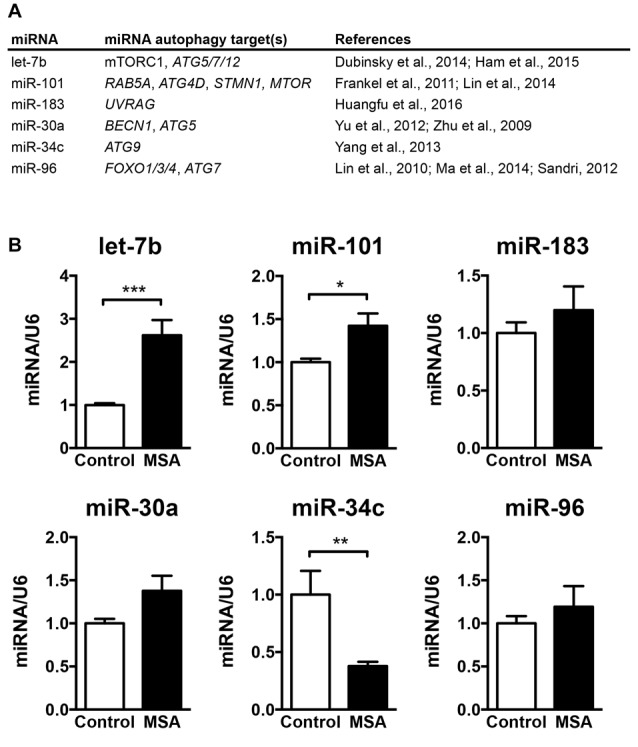
Levels of microRNAs (miRNAs) regulating the expression of autophagy proteins in the striatum of multiple system atrophy (MSA) cases. **(A)** Summary of the miRNAs analyzed by quantitative PCR (qPCR) in MSA brains, together with their reported autophagy targets. **(B)** qPCR analysis of the relative levels of let-7b, miR-101, miR-183, miR-30a, miR-34c and miR-96 in striatum of healthy controls and MSA patients. Results are expressed as averages ± SEM. Statistical analysis was performed by Student’s *t*-test. *p < 0.05, **p < 0.01, ***p < 0.001.

The main goal of this study was to investigate the role of miRNAs in biological processes associated to the MSA disease pathology, such as autophagy. Therefore, we selected a group of miRNAs that reportedly modulate the expression of autophagy genes, including the miRNAs let-7b (Dubinsky et al., 2014; Ham et al., 2015), miR-101 (Frankel et al., 2011; Lin et al., 2014), miR-183 (Huangfu et al., 2016), miR-30a (Zhu et al., 2009; Yu et al., 2012), miR-34c (Yang et al., 2013) and miR-96 (Lin et al., 2010; Sandri, 2012; Ma et al., 2014; Figure 1A). We analyzed the relative expression levels of these autophagy-regulating miRNAs in the striatum (caudate nucleus and putamen) of MSA-P cases obtained from three different institutions across the United States (UCSD-ADRC, Johns Hopkins Medical Institution, and Banner Sun Health Research Institute; Supplementary Table S1). Striatum was selected as it is a region severely affected by neurodegeneration in the MSA-P pathology (Gilman et al., 2008). By qPCR analysis, we observed a significant increase in let-7b and miR-101, a reduction in miR-34c, and a trend for higher levels of miR-183, miR-30a and miR-96 in the striatum of MSA patients (Figure 1B). Interestingly, changes in miRNA levels were specifically localized in the striatum, and no significant miRNA changes were observed in frontal cortex or cerebellum, with the exception of a reduction of miR-30a in frontal cortex (Supplementary Figure S1).

**Figure 2:**
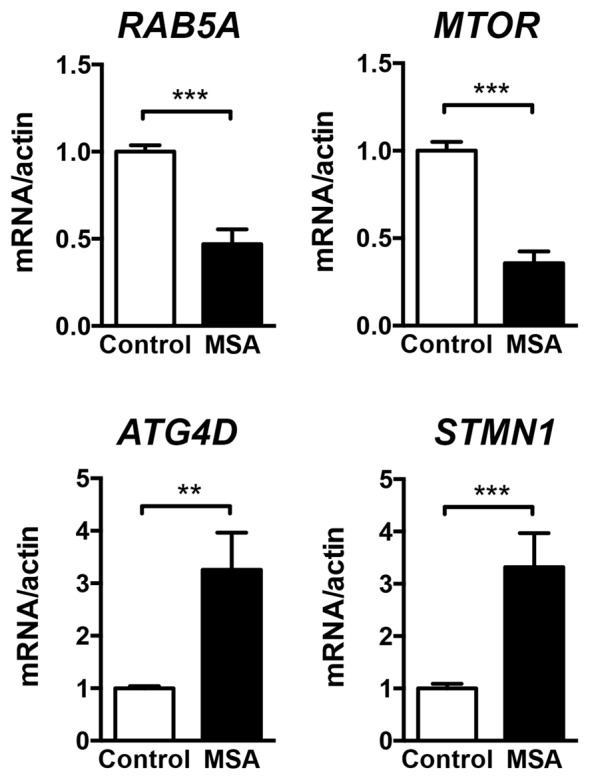
Expression of miR-101-regulated genes in the striatum of MSA cases. qPCR analysis of the relative mRNA levels of *RAB5A*, *MTOR*, *ATG4D* and *STMN1* in the striatum of healthy controls and MSA patients. Results are expressed as averages ± SEM. Statistical analysis was performed by Student’s *t*-test. **p < 0.01, ***p < 0.001.

For a more in-depth study of the mechanistic relationship between miRNA changes and deficits in protein clearance in the MSA brain, we decided to focus in miR-101, as alterations in this miRNA have been strongly associated with autophagy impairments in human cancer models (Frankel et al., 2011; Xu et al., 2013). These reports also identified *RAB5A* as one of the most relevant targets for miR-101 in humans (miRNA support vector regression (mirSVR) score = −3.47; Betel et al., 2010). The protein Rab5a is involved in the sorting of endocytic vesicles towards autophagy degradation, and inhibition of its expression might lead to protein clearance impairments (Wu et al., 2015). Other miR-101 autophagy targets include *MTOR* (mirSVR = −0.82; Lin et al., 2014), and the lower affinity targets *ATG4D* (mirSVR = −0.73) and *STMN1* (Xu et al., 2013). The mRNA levels of *RAB5A* and *MTOR* were significantly down-regulated in the striatum of MSA patients (Figure 2), consistent with the significant increase in miR-101 detected in this region. However, the expression of miR-101 target genes *ATG4D* and *STMN1* was increased in striatum (Figure 2), suggesting that other mechanisms may be involved in the regulation of their expression. Finally, the relative mRNA levels of the α-syn gene (*SNCA*) were significantly increased in striatum (Supplementary Figure S2), and we observed a positive correlation between miR-101 levels and *SNCA* expression in this brain region (Pearson’s *r* = 0.6684, *p* < 0.01), suggesting a potential mechanistic relationship between α-syn pathology and miR-101 levels.

### Autophagy Is Inhibited in the Striatum of MSA Patients

**Figure 3:**
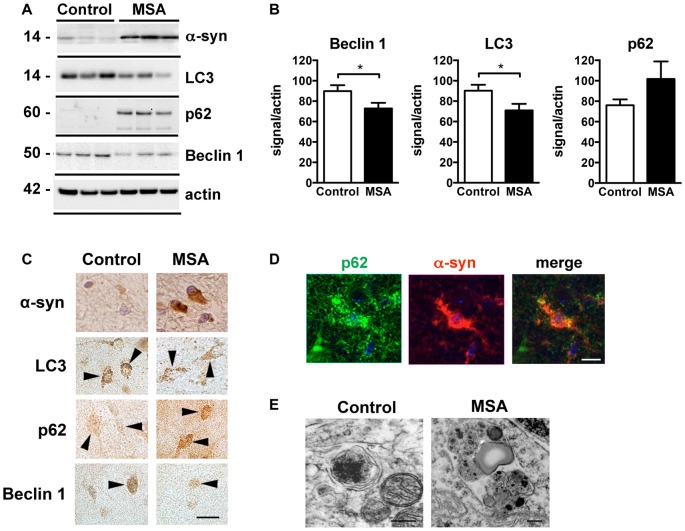
Autophagy impairments in the striatum of MSA cases. **(A)** Representative immunoblot results of alpha-synuclein (α-syn), Beclin 1, LC3 and p62 in protein homogenates from the striatum of healthy controls and MSA patients. **(B)** Densitometric quantification of immunopositive Beclin 1, LC3 and p62 bands in protein homogenates from the striatum of healthy controls and MSA patients. Statistical analysis was performed by Student’s *t*-test. *p < 0.05. **(C)** Representative images of immunohistochemical staining for α-syn, Beclin 1, LC3 and p62 in the striatum of healthy controls and MSA patients. Arrowheads highlight immunopositive cells. Scale bar, 10 μm. **(D)** Representative immunofluorescence images of an oligodendroglial cell showing p62 (green) and α-syn (red) co-localization in the striatum of MSA patient. Scale bar, 5 μm. **(E)** Representative electron microscopy images of oligodendroglial cells showing altered morphology of autophagosomes in the striatum of a healthy control and a MSA patient. Scale bar, 250 nm.

Deficits in autophagy clearance have been reported in MSA and in other neurodegenerative diseases such as PD, DLB and AD (Cuervo et al., 2004; Pickford et al., 2008; Crews et al., 2010). In order to validate those reported autophagy deficits in our brain samples, we analyzed the levels of the autophagy proteins Beclin 1, LC3 and p62 in striatum samples of control and MSA patients by immunoblot (Figures 3A,B). We observed a decrease in the levels of the autophagy proteins Beclin 1 and LC3, and p62 appeared elevated, suggestive of autophagy impairments (Figures 3A,B). Consistent with previous reports showing low levels of LC3-II in protein homogenates from human brain tissue (Klionsky et al., 2016), we observed a weak LC3-II signal in our samples (Supplementary Figure S3). Confocal microscopy analysis confirmed the immunoblot results (Figure 3C), and showed that p62 co-localized with α-syn accumulation in the striatum of MSA patients (Figure 3D). Moreover, visualization of autophagosomes by electron microscopy revealed that, while in brain cells from healthy patients autophagosomes are small and contain electrodense material, in MSA brains they are bigger in size, and present higher number of electrodense inclusions and lipidic granules (Figure 3E). These results confirm the presence of autophagy deficits in the striatum of MSA patients.

### Lentiviral Delivery of miR-101 Leads to Autophagy Inhibition in the Oligodendroglial Cell Line CG-4

**Figure 4:**
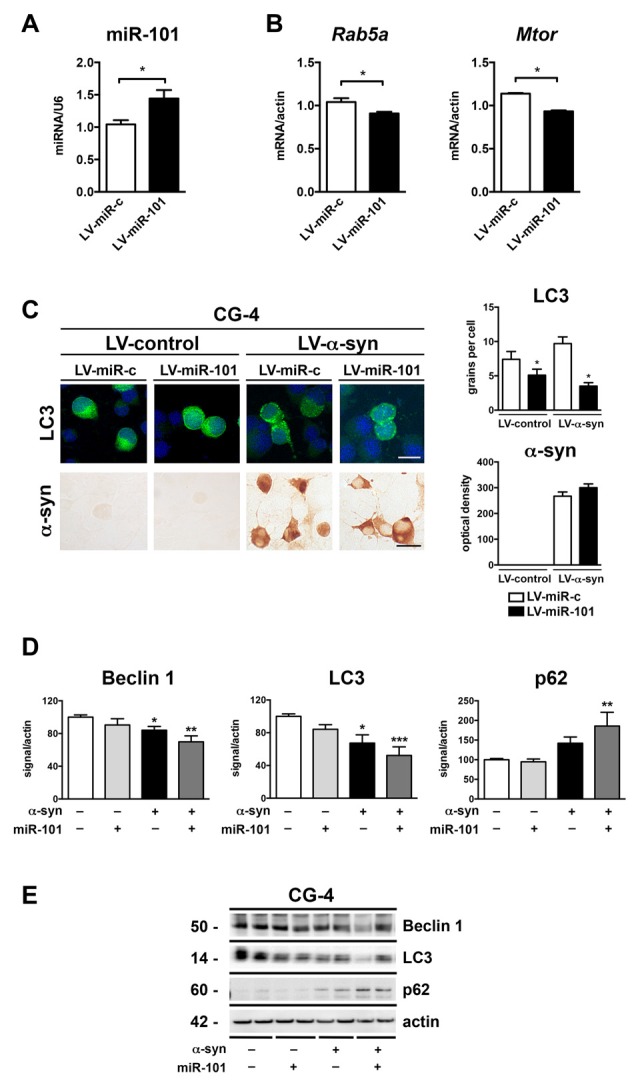
Overexpression of miR-101 inhibits autophagy and induces α-syn accumulation in the oligodendroglial cell line CG-4. **(A)** qPCR analysis of the levels of miR-101 following infection with a lentivirus (LV) control (LV-miR-c) or a LV expressing miR-101a (LV-miR-101) in CG-4 cells. **(B)** qPCR analysis of the levels of *Rab5a* and *Mtor* mRNAs following infection with LV-miR-c or LV-miR-101 in CG-4 cells. Results are expressed as averages ± SEM. Statistical analysis was performed by Student’s *t*-test. *p < 0.05. **(C)** Immunocytochemical analysis of CG-4 cells co-infected with LV-control or LV-α-syn, and with LV-miR-c or LV-miR-101. miR-101 expression was monitored using the fluorescent reporter GFP (not shown). A LV expressing LC3-GFP was used to assess intracellular LC3 levels, and quantification is presented as the average number of LC3 positive granules per cell. Immunostaining with an anti-α-syn antibody was used to determine α-syn immunoreactivity, and quantification results are presented as optical density units. Results are expressed as averages ± SEM. Statistical analysis was performed by one-way analysis of variance (ANOVA) with Tukey’s *post hoc* test. *p < 0.05 when compared to the double control condition. **(D)** Densitometry quantification of immunoblots for Beclin 1, LC3 and p62 in homogenates of CG-4 cells co-infected with LV-control or LV-α-syn, and LV-miR-c or LV-miR-101. **(E)** Representative immunoblot results of α-syn, Beclin 1, LC3 and p62 in cell homogenates of CG-4 cells co-infected with LV-control or LV-α-syn, and LV-miR-c or LV-miR-101. Results are expressed as averages ± SEM. Statistical analysis was performed by one-way ANOVA with Tukey’s *post hoc* test. *p < 0.05, **p < 0.01, ***p < 0.001 when compared to the double control condition.

To evaluate whether alterations in miR-101 levels lead to autophagy deficits and α-syn accumulation in oligodendrocytes, we performed *in vitro* experiments using the oligodendroglial cell line CG-4 (Louis et al., 1992). Transduction of CG-4 cells with a lentiviral construct expressing rat miR-101 (LV-miR-101) effectively increased miR-101 levels in comparison to cells expressing a control vector (LV-miR-control; Figure 4A). Importantly, transcription of two predicted targets of miR-101, *Rab5a* (mirSVR = −1.1) and *Mtor* (mirSVR = −0.2), was significantly reduced in LV-miR-101-infected CG-4 cells (Figure 4B), suggesting that this epigenetic mechanism is active in oligodendrocytes.

We next co-infected CG-4 cells with a LV expressing human α-syn (LV-α-syn) or its control vector (LV-control); with a LV-miR-101 or LV-miR-control; and with LV-LC3-GFP to monitor autophagy status. Co-infection with LV-α-syn and LV-miR-101 induced a significant reduction in the LC3 signal, but not a significant increase in α-syn accumulation as measured by immunocytochemistry (Figure 4C). The effect of miR-101 overexpression on autophagy was confirmed by immunoblot analysis of the autophagy proteins Beclin 1, LC3 and p62 in cell extracts from CG-4 cells co-expressing α-syn and miR-101 (Figures 4D,E). Co-infection with α-syn and miR-101 induced a significant decrease in Beclin 1 and LC3 protein levels, and a significant increase in p62 in CG-4 cells when compared to the control condition (Figures 4D,E), worsening the effects of α-syn alone. Taken together, our results indicate that miR-101 has a modulatory effect on autophagy in oligodendroglial cells, however we cannot rule out the possibility of this miRNA also inhibiting other protein clearance mechanisms.

### Lentiviral Delivery of an antimiR-101 Construct Reduces α-syn-Induced Autophagy Deficits in the Oligodendroglial Cell Line CG-4

**Figure 5:**
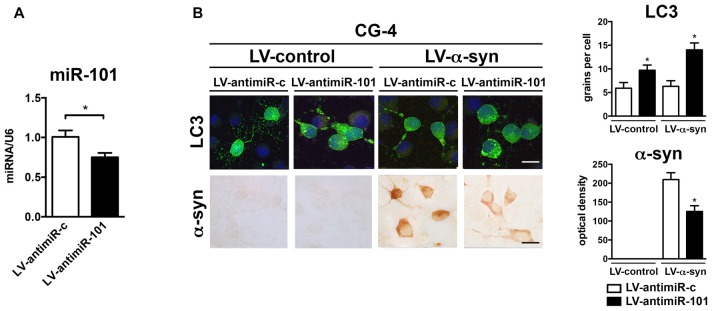
Overexpression of antimiR-101 induces autophagy and reduces α-syn accumulation in the oligodendroglial cell line CG-4. **(A)** qPCR analysis of the levels of miR-101 following infection with a LV control (LV-antimiR-c) or a LV expressing antimiR-101a (LV-antimiR-101) in CG-4 cells. Results are expressed as averages ± SEM. Statistical analysis was performed by Student’s *t*-test. *p < 0.05. **(B)** Immunocytochemical analysis of CG-4 cells co-infected with LV-control or LV-α-syn, and with LV-antimiR-c or LV-antimiR-101. AntimiR-101 expression was monitored using the fluorescent reporter mCherry (not shown). A LV expressing LC3-GFP was used to assess intracellular LC3 levels, and quantification is presented as the average number of LC3 positive granules per cell. Immunostaining with an anti-α-syn antibody was used to determine α-syn immunoreactivity, and quantification results are presented as optical density units. Results are expressed as averages ± SEM. Statistical analysis was performed one-way ANOVA with Tukey’s *post hoc* test. *p < 0.05 when compared to the double control condition.

To confirm the mechanistic involvement of miR-101 in oligodendroglial autophagy, CG-4 were infected with a lentiviral construct expressing an antisense sequence against miR-101 (LV-antimiR-101; Figure 5). Three days after infection, we observed a significant reduction in miR-101 levels when compared to antimiR-control (LV-antimiR-c; Figure 5A). Interestingly, transcription of either *Rab5a* or *Mtor* was not significantly altered by antimiR-101 overexpression (not shown), which may be an indication that further reducing already low levels of endogenous miR-101 is not sufficient to significantly alter the expression of these genes. CG-4 cells were then co-infected with LV-α-syn and LV-antimiR-101 (or controls), and with LV-LC3-GFP to monitor autophagy by immunocytochemistry (Figure 5B). Co-infection with LV-α-syn and LV-antimiR-101 induced an increase in the LC3 signal, and a significant decrease in the intracellular accumulation of α-syn in CG-4 cells, suggesting that the repression of miR-101 may effectively induce α-syn clearance *in vitro* (Figure 5B). The miR-101 target genes directly involved in the autophagy enhancing effects of antimiR-101 remain to be investigated.

### Lentiviral Delivery of an antimiR-101 Construct Reduces α-syn-Induced Autophagy Deficits in the MBP-α-syn Transgenic Mouse Model of MSA

**Figure 6:**
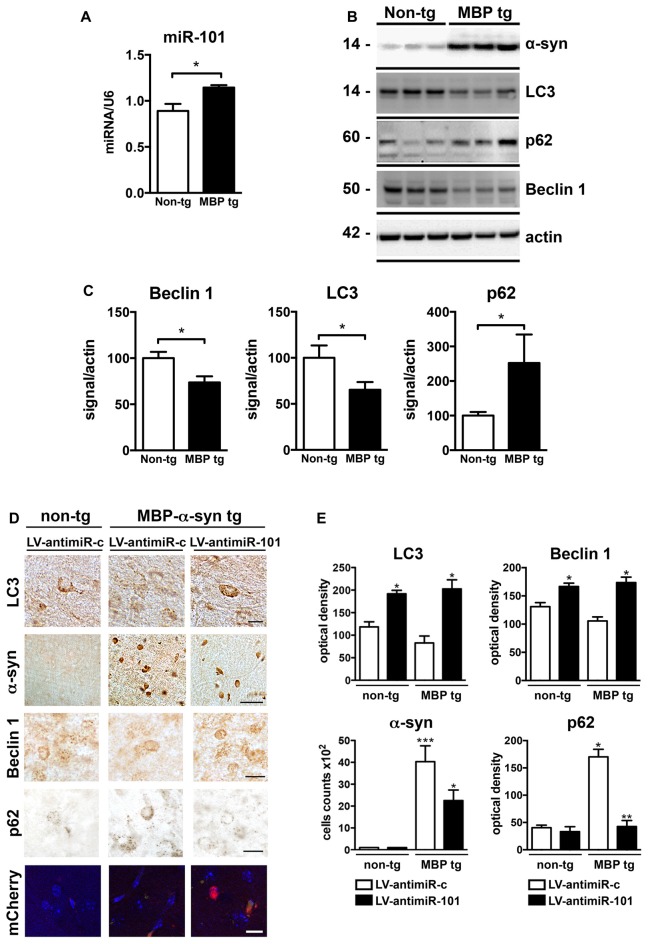
Expression of antimiR-101 induces autophagy and reduces α-syn accumulation in myelin basic protein (MBP)-α-syn transgenic (tg) mouse brains. **(A)** qPCR analysis of the relative levels of miR-101 in the striatum of non-tg and MBP-α-syn tg mice. **(B)** Representative immunoblot results of α-syn, Beclin 1, LC3 and p62 in brain homogenates of non-tg and MBP-α-syn tg mouse brains. **(C)** Densitometric quantification of immunopositive Beclin 1, LC3 and p62 bands in brain homogenates of non-tg and MBP-α-syn tg mice. Results are expressed as averages ± SEM. Statistical analysis was performed by Student’s *t*-test. *p < 0.05. **(D)** Lentiviral constructs expressing control vector (LV-antimiR-c) or antimiR-101 (LV-antimiR-101) were injected bilaterally in the striatum of MBP-α-syn tg, and effects analyzed 6 weeks later by immunohistochemistry. Representative images of immunohistochemical staining for LC3, α-syn, Beclin 1 and p62 in the striatum of non-tg and MBP-α-syn tg mice injected with LV-antimiR-c or LV-antimiR-101. Scale, 50 μm for α-syn, 5 μm for LC3 and antimiR-101 reporter (mCherry). **(E)** Optical density quantification of LC3, Beclin 1 and p62 immunostaining and cell counts per field of α-syn positive cells in the striatum of non-tg and MBP-α-syn tg mice injected with LV-antimiR-c or LV-antimiR-101. AntimiR-101 expression was monitored using a fluorescent reporter. Results are expressed as averages ± SEM. Statistical analysis was performed by one-way ANOVA with Tukey’s *post hoc* test. ***p < 0.001; **p < 0.01; *p < 0.05 when compared to non-tg animals injected with LV-antimiR-c.

We next investigated the role of miR-101 in the brains of MBP-α-syn transgenic (tg) mice, a mouse model of MSA. MBP-α-syn mice express human α-syn under the control of the oligodendroglial MBP promoter, and show α-syn accumulation in striatum and other brain areas, accompanied by neuroinflammation and motor deficits (Shults et al., 2005; Valera et al., 2014). We observed a significant increase in the levels of miR-101 in the striatum of MBP-α-syn tg mice (Line 29, high α-syn expresser) when compared to non-tg controls (Figure 6A). We also observed a significant increase in the levels of miR-183 and miR-96 (Supplementary Figure S4). These results confirm not only the potential involvement of miR-101 in the oligodendroglial pathology of the striatum in MSA-tg mice (as these animals overexpress human α-syn exclusively in oligodendrocytes), but they also suggest that increased levels of miR-183 and miR-96 in the same brain region might cooperate to further inhibit autophagy in this mouse model. In agreement with these results, autophagy proteins Beclin 1 and LC3 were significantly decreased, while p62 levels were increased in MBP-α-syn tg brains when compared to non-tg mice (Figures 6B,C). As observed by immunofluorescence, the autophagy proteins Beclin 1, LC3 and p62 co-localized with α-syn and with the oligodendroglial marker Olig2 in the striatum of MBP-α-syn tg mice (Supplementary Figure S5). However, mRNA levels of the miR-101 predicted targets *Rab5a* (mirSVR = −1.12) and *Mtor* (mirSVR = −0.38) showed no significant changes in striatum (not shown). This may be due to the low predicted affinity of miR-101 for these targets.

To confirm that miR-101 is contributing to autophagy dysregulation *in vivo*, a proof-of-concept experiment was performed in which we delivered LV expressing antimiR-101 by bilateral stereotaxic injection into the striatum of non-tg and MBP-α-syn tg mice, and compared to animals injected with LV-antimiR-control (Figures 6D,E). For this experiment, we used the Line 1, a milder α-syn expresser, as the severe pathology exhibited by Line 29 animals limit interventions. Six weeks after LV injections, non-tg and MBP-α-syn brains were sectioned and analyzed by immunohistochemistry (Figure 6D). LC3 levels were diminished in the striatum of MBP-α-syn tg mice, consistent with the reported impairment in autophagy, and antimiR-101 expression significantly increased LC3 levels in both non-tg and tg animals (Figure 6E). Moreover, the number of cells accumulating α-syn in striatum was reduced after antimiR-101 delivery (Figure 6E), suggesting a potential disease-modifying effect of the antimiR-101 therapy. Additionally, Beclin 1 levels were increased by antimiR-101 delivery in both non-tg and tg animals, while p62 levels were significantly decreased in MBP-α-syn tg brains following antimiR-101 treatment (Figures 6D,E). These results serve as a proof of concept that warrants further evaluation of alternative interventions targeting miR-101 for the potential treatment of MSA or related disorders with autophagy impairment.

## Discussion

In this report, we present evidence supporting the involvement of miR-101 in the alteration of autophagy observed in MSA brains, that contributes to α-syn accumulation. We observed that overexpression of miR-101 inhibited autophagy in oligodendrocytes, while expression of an antimiR-101 construct alleviated some of these autophagy deficits *in vitro* and *in vivo* in a mouse model of MSA. Our results suggest that miR-101, alone or together with other miRNAs, might represent an additional inhibitory mechanism of the protein clearance machinery contributing to pathological α-syn accumulation. Consequently, therapies targeting autophagy-regulating miRNAs such as miR-101 might have beneficial effects on MSA patients.

Changes in the miRNA expression profile have been observed in numerous neurodegenerative disorders (Lee et al., 2011; Cardo et al., 2014; Qiu et al., 2014; Vallelunga et al., 2014; Denk et al., 2015; Femminella et al., 2015; Tan et al., 2015; Hoss et al., 2016), and those changes were initially explored as a potential biomarker alternative for their differential or early diagnosis. However, evidence has recently mounted suggesting that miRNA changes might also be mechanistically linked to the molecular origin and progression of the disease. This is the case of miR-30a and miR-124, which have been associated with the pathological changes observed in AD, PD and Huntington’s disease (Mellios et al., 2008; Fang et al., 2012; Lang et al., 2012; Sun et al., 2015). In a previous study, we identified specific miRNAs that are upregulated in MSA using microarray profiling in postmortem brain samples (Ubhi et al., 2014). Interestingly, 95% of dysregulated miRNAs were up-regulated in MSA cases, while only 5% were down-regulated when compared to non-diseased controls, suggesting that miRNA-mediated gene silencing might be a prominent feature in MSA. Furthermore, the miRNA maturation machinery was preserved in both MSA patients and animal models (Ubhi et al., 2014), indicating that miRNA changes are probably due to deregulated expression and/or to degeneration of selected cell populations. In this study, we have performed a more in-depth analysis taking into consideration biological processes that have been reported as altered in MSA brains, such as autophagy (Schwarz et al., 2012). We found that miRNAs that reportedly modulate the expression of autophagy genes are up-regulated in MSA brains and MSA models, suggesting that pathological miRNA changes could constitute an additional mechanism inhibiting autophagy in MSA.

Our results show that there is an important regional component regarding miRNA changes in MSA. We observed specific increase of five out of six selected miRNAs in the striatum of MSA brains, and these changes were not evident in frontal cortex or cerebellum samples. Interestingly, miR-34c was significantly reduced in striatum, in line with recent studies showing that miR-34 mitigates neurodegeneration (Liu et al., 2012), and inhibition of miR-34c enhances α-synuclein expression in PD (Kabaria et al., 2015). We found a significant increase in let-7b, and a trend for higher levels of miR-183, miR-30a and miR-96 in MSA striatum. miR-183 regulates autophagy and apoptosis in colorectal cancer through targeting of the ultraviolet radiation resistance-associated gene (*UVRAG*), a well-known regulator of autophagy that promotes autophagosome formation and maturation (Huangfu et al., 2016). While it has been reported that let-7 coordinately suppresses components of the amino acid sensing pathway to repress mTORC1 and induce autophagy (Dubinsky et al., 2014), it has also been shown that let-7b suppresses apoptosis and autophagy in human mesenchymal stem cells (Ham et al., 2015). miR-30a acts as post-transcriptional inhibitor of BDNF in prefrontal cortex (Mellios et al., 2008), suggesting that the consequences of pathological miRNA up-regulation in MSA may not be limited to autophagy, and rather contributing with other neurodegenerative pathways. Finally, miR-96 has been reported to target *ATG7* or *MTOR* for inhibition depending on its expression levels, resulting in a bi-phasic effect that can either inhibit or promote autophagy (Ma et al., 2014). It can be concluded that the miRNA regulation of physiological cellular processes is a complex epigenetic mechanism, and that the balance between opposing signals (activation vs. repression), and potentially the cross-talk with other regulatory pathways, may be critical to determine the outcome of the observed miRNA changes.

We focused our study on miR-101 due to its reported potent inhibitory effect on autophagy (Frankel et al., 2011), and because it showed consistent and significant increased levels in MSA striatum samples. However, we cannot rule out the possibility of other miRNAs, or more likely a combination of miRNAs, contributing to the autophagy dysfunction in MSA brains. Results linking miR-101 to autophagy inhibition were first reported in breast cancer cells (Frankel et al., 2011), and later reproduced in hepatocellular carcinoma cells (Xu et al., 2013) and cardiomyocytes (Wu et al., 2015). Three of the autophagy targets of miR-101 (*RAB5A*, *ATG4D* and *STMN1*) have been validated in human cancer models (Frankel et al., 2011; Jing et al., 2015). Another reported miR-101 target in the autophagy pathway is *MTOR* (Xu et al., 2013; Lin et al., 2014), an autophagy inhibitor suggesting that miR101 is regulating both autophagy inhibitors and activators with an overall balance toward blocking autophagy. In agreement with these studies, we provide evidence of similar regulation in the brain where we observed a decrease in *RAB5A* and *MTOR* expression along with decreased BECLIN1 and LC3 autophagy markers in association with an increase in miR-101 in striatum. These results further substantiate our rationale for exploring the involvement of altered levels of miR-101 in the autophagy deficits observed in neurodegenerative disorders such as MSA. However, the expression of the lower mirSVR score targets *ATG4D* and *STMN1* was increased in striatum, suggesting that other non-miR-101 factors are at play in the regulation of autophagy in MSA in the striatum. In this sense, it is important to note that some reports have shown that the autophagy-lysosomal pathway is activated in the pons of MSA patients (Makioka et al., 2012; Schwarz et al., 2012), suggesting an additional layer of complexity to region-specific regulatory mechanism(s). Interestingly, miR-101 is also a negative regulator of amyloid precursor protein expression and modulates the accumulation of amyloid beta in hippocampal neurons (Vilardo et al., 2010; Barbato et al., 2014), highlighting a potential role for miR-101 in other neuropathological conditions through additional mechanisms.

Our results suggest that striatum is affected by changes in miRNAs that regulate autophagy genes in MSA. Importantly, neurodegeneration is severe in the striatum (caudate/putamen) and substantia nigra of MSA-P patients (Jellinger, 2014). Moreover, the extent and spatial distribution of functional and morphological changes in the striatum enables the differentiation of MSA-P from PD (Ghaemi et al., 2002). A positive association has been reported between striatal dopamine loss and α-syn accumulation in the striatum of MSA patients, but not in substantia nigra (Tong et al., 2010). These reports highlight the importance of striatal changes in MSA pathology, and together with our results suggest that this brain region could be the most sensitive area to miRNA-targeted therapeutics for MSA.

Our proof-of-concept experiment using brain region-specific overexpression of an antimiR-101 construct suggests that miRNA-targeted approaches may be an option for the treatment of MSA and related disorders. As mentioned, for this experiment we decided to use a milder α-syn expresser line (Line 1), as the Line 29 animals exhibit severe pathology that drastically limits survivability to invasive interventions. Despite the fact that Line 1 animals do not show significant changes in striatal miR-101 levels, they can be used as a model for testing early disease interventions. Interestingly, the expression levels of the miR-101 predicted targets *Rab5a* and *Mtor* showed no significant changes in the striatum of MBP-α-syn tg mice, despite the significant increase in miR-101 levels. To interpret these results, it is important to consider that MBP-α-syn tg mice overexpress α-syn exclusively in oligodendrocytes, while mRNA changes were measured in whole brain, thus potentially attenuating cell type-specific effects of miRNA changes on the expression of autophagy genes.

Additional research will be required to address the question of how anti-miRNA therapies ameliorate oligodendroglial vs. neuronal α-syn accumulation in MSA models. Recently, a new hypothesis has gained momentum suggesting that MSA neurons release toxic aggregates of α-syn to the extracellular medium, and this extracellular, propagating α-syn is incorporated and accumulated by oligodendrocytes, a cell type that arguably expresses lower levels of endogenous α-syn (Asi et al., 2014). In this sense, α-syn would behave as a prion-like molecule (Prusiner et al., 2015), and its spreading behavior might be due to the inability of the acceptor cells to efficiently clear α-syn by intrinsic mechanisms such as autophagy. Although we have observed that miR-101 may play a role in oligodendroglial autophagic dysfunction, miR-101 dysregulation might also be altering the pattern of neuronal α-syn accumulation and its release to the extracellular environment. Experiments assessing the effect of antimiR-101 on α-syn propagation will be needed to further evaluate its potential as a MSA therapeutic. Finally, autophagy is a highly-regulated process and its inhibition is most likely due to a combination of factors and not only to a miRNA imbalance. This is specifically relevant in the case of neurodegenerative diseases, in which numerous genetic and environmental factors combine with natural aging processes to result in neurodegeneration. Therefore, the success of anti-miRNA approaches against miRNAs regulating autophagy will depend on the balance of miRNA regulation with other autophagy inhibiting and pro-neurodegenerative processes. In this sense, we hypothesize that combining an antimiR-101 approach with additional α-syn anti-aggregation therapies may lead to significant beneficial effects for MSA patients.

In conclusion, our results suggest that miRNA dysregulation contributes to MSA pathology, with miR-101 alterations partially mediating autophagy impairment. Therefore, therapies targeting miR-101 may represent potential approaches for MSA and related neuropathologies with autophagy dysfunction.

## Author Contributions

EV, PD and EM conceived the study and participated in its design. EV, BS, JM, MT, AA, MM, ER and EM performed the experiments. JT, TB and EM provided human tissue samples. EV, BS, JCT, TGB, PD and EM wrote the article. All authors read and approved the final manuscript.

## Conflict of Interest Statement

The authors declare that the research was conducted in the absence of any commercial or financial relationships that could be construed as a potential conflict of interest.

## Abbreviations

α-syn: alpha-synuclein
AD: Alzheimer’s disease
ANOVA: analysis of variance
antimiR-101: anti-microRNA-101
CNS: central nervous system
DLB: dementia with Lewy bodies
LV: lentivirus
miR-101: microRNA-101
miRNA: microRNA
mirSVR: microRNA support vector regression
MBP: myelin basic protein
MSA: multiple system atrophy
PD: Parkinson’s disease
qPCR: quantitative PCR
tg: transgenic.

## References

[B1] Alvarez-Erviti L. Seow Y. Schapira A. H. Rodriguez-Oroz M. C. Obeso J. A. Cooper J. M. (2013). Influence of microRNA deregulation on chaperone-mediated autophagy and α-synuclein pathology in Parkinson’s disease. Cell Death Dis. 4:e545. 10.1038/cddis.2013.73 23492776 PMC3615743

[B2] Asi Y. T. Simpson J. E. Heath P. R. Wharton S. B. Lees A. J. Revesz T.. (2014). α-synuclein mRNA expression in oligodendrocytes in MSA. Glia 62, 964–970. 10.1002/glia.22653 24590631 PMC4238782

[B3] Banzhaf-Strathmann J. Beinto E. May S. Arzberger T. Tahirovic S. Kretzschmar H.. (2014). MicroRNA-125b induces tau hyperphosphorylation and cognitive deficits in Alzheimer’s disease. EMBO J. 33, 1667–1680. 10.15252/embj.201387576 25001178 PMC4194100

[B4] Barbato C. Pezzola S. Caggiano C. Antonelli M. Frisone P. Ciotti M. T.. (2014). A lentiviral sponge for miR-101 regulates RanBP9 expression and amyloid precursor protein metabolism in hippocampal neurons. Front. Cell. Neurosci. 8:37. 10.3389/fncel.2014.00037 24592211 PMC3923151

[B5] Bar-On P. Crews L. Koob A. O. Mizuno H. Adame A. Spencer B.. (2008). Statins reduce neuronal α-synuclein aggregation in *in vitro* models of Parkinson’s disease. J. Neurochem. 105, 1656–1667. 10.1111/j.1471-4159.2008.05254.x 18248604 PMC2822545

[B6] Beach T. G. Adler C. H. Sue L. I. Serrano G. Shill H. A. Walker D. G.. (2015). Arizona study of aging and neurodegenerative disorders and brain and body donation program. Neuropathology 35, 354–389. 10.1111/neup.12189 25619230 PMC4593391

[B7] Betel D. Koppal A. Agius P. Sander C. Leslie C. (2010). Comprehensive modeling of microRNA targets predicts functional non-conserved and non-canonical sites. Genome Biol. 11:R90. 10.1186/gb-2010-11-8-r90 20799968 PMC2945792

[B8] Birdsill A. C. Walker D. G. Lue L. Sue L. I. Beach T. G. (2011). Postmortem interval effect on RNA and gene expression in human brain tissue. Cell Tissue Bank. 12, 311–318. 10.1007/s10561-010-9210-8 20703815 PMC3343031

[B9] Cardo L. F. Coto E. Ribacoba R. Menéndez M. Moris G. Suárez E.. (2014). MiRNA profile in the substantia nigra of Parkinson’s disease and healthy subjects. J. Mol. Neurosci. 54, 830–836. 10.1007/s12031-014-0428-y 25284245

[B10] Crews L. Spencer B. Desplats P. Patrick C. Paulino A. Rockenstein E.. (2010). Selective molecular alterations in the autophagy pathway in patients with Lewy body disease and in models of α-synucleinopathy. PLoS One 5:e9313. 10.1371/journal.pone.0009313 20174468 PMC2824828

[B11] Cuervo A. M. Stefanis L. Fredenburg R. Lansbury P. T. Sulzer D. (2004). Impaired degradation of mutant α-synuclein by chaperone-mediated autophagy. Science 305, 1292–1295. 10.1126/science.1101738 15333840

[B12] Denk J. Boelmans K. Siegismund C. Lassner D. Arlt S. Jahn H. (2015). MicroRNA profiling of CSF reveals potential biomarkers to detect Alzheimer’s disease. PLoS One 10:e0126423. 10.1371/journal.pone.0126423 25992776 PMC4439119

[B13] Desplats P. Lee H. J. Bae E. J. Patrick C. Rockenstein E. Crews L.. (2009). Inclusion formation and neuronal cell death through neuron-to-neuron transmission of α-synuclein. Proc. Natl. Acad. Sci. U S A 106, 13010–13015. 10.1073/pnas.0903691106 19651612 PMC2722313

[B14] Desplats P. Patel P. Kosberg K. Mante M. Patrick C. Rockenstein E.. (2012). Combined exposure to Maneb and Paraquat alters transcriptional regulation of neurogenesis-related genes in mice models of Parkinson’s disease. Mol. Neurodegener. 7:49. 10.1186/1750-1326-7-49 23017109 PMC3502617

[B15] Dickson D. W. Lin W.-L. Liu W.-K. Yen S.-H. (1999a). Multiple system atrophy: a sporadic synucleinopathy. Brain Pathol. 9, 721–732. 10.1111/j.1750-3639.1999.tb00553.x 10517510 PMC8098455

[B16] Dickson D. W. Liu W.-K. Hardy J. Farrer M. Mehta N. Uitti R.. (1999b). Widespread alterations of α-synuclein in multiple system atrophy. Am. J. Pathol. 155, 1241–1251. 10.1016/S0002-9440(10)65226-1 10514406 PMC1867032

[B17] Dubinsky A. N. Dastidar S. G. Hsu C. L. Zahra R. Djakovic S. N. Duarte S.. (2014). Let-7 coordinately suppresses components of the amino acid sensing pathway to repress mTORC1 and induce autophagy. Cell Metab. 20, 626–638. 10.1016/j.cmet.2014.09.001 25295787 PMC4245205

[B18] Fang M. Wang J. Zhang X. Geng Y. Hu Z. Rudd J. A.. (2012). The miR-124 regulates the expression of BACE1/β-secretase correlated with cell death in Alzheimer’s disease. Toxicol. Lett. 209, 94–105. 10.1016/j.toxlet.2011.11.032 22178568

[B19] Femminella G. D. Ferrara N. Rengo G. (2015). The emerging role of microRNAs in Alzheimer’s disease. Front. Physiol. 6:40. 10.3389/fphys.2015.00040 25729367 PMC4325581

[B20] Frankel L. B. Lund A. H. (2012). MicroRNA regulation of autophagy. Carcinogenesis 33, 2018–2025. 10.1093/carcin/bgs266 22902544

[B21] Frankel L. B. Wen J. Lees M. Høyer-Hansen M. Farkas T. Krogh A.. (2011). microRNA-101 is a potent inhibitor of autophagy. EMBO J. 30, 4628–4641. 10.1038/emboj.2011.331 21915098 PMC3243595

[B22] Fu L.-L. Wen X. Bao J. K. Liu B. (2012). MicroRNA-modulated autophagic signaling networks in cancer. Int. J. Biochem. Cell Biol. 44, 733–736. 10.1016/j.biocel.2012.02.004 22342941

[B23] Fullgrabe J. Klionsky D. J. Joseph B. (2014). The return of the nucleus: transcriptional and epigenetic control of autophagy. Nat. Rev. Mol. Cell Biol. 15, 65–74. 10.1038/nrm3716 24326622

[B24] Gascon E. Gao F. B. (2014). The emerging roles of microRNAs in the pathogenesis of frontotemporal dementia-amyotrophic lateral sclerosis (FTD-ALS) spectrum disorders. J. Neurogenet. 28, 30–40. 10.3109/01677063.2013.876021 24506814 PMC4199862

[B25] Ghaemi M. Hilker R. Rudolf J. Sobesky J. Heiss W. D. (2002). Differentiating multiple system atrophy from Parkinson’s disease: contribution of striatal and midbrain MRI volumetry and multi-tracer PET imaging. J. Neurol. Neurosurg. Psychiatry 73, 517–523. 10.1136/jnnp.73.5.517 12397143 PMC1738131

[B26] Gilman S. Wenning G. K. Low P. A. Brooks D. J. Mathias C. J. Trojanowski J. Q.. (2008). Second consensus statement on the diagnosis of multiple system atrophy. Neurology 71, 670–676. 10.1212/01.WNL.0000324625.00404.15 18725592 PMC2676993

[B27] Ham O. Lee S.-Y. Lee C. Y. Park J.-H. Lee J. Seo H.-H.. (2015). let-7b suppresses apoptosis and autophagy of human mesenchymal stem cells transplanted into ischemia/reperfusion injured heart 7by targeting caspase-3. Stem Cell Res. Ther. 6:147. 10.1186/s13287-015-0134-x 26296645 PMC4546263

[B28] Hoss A. G. Labadorf A. Beach T. G. Latourelle J. C. Myers R. H. (2016). microRNA profiles in Parkinson’s disease prefrontal cortex. Front. Aging Neurosci. 8:36. 10.3389/fnagi.2016.00036 26973511 PMC4772525

[B29] Huangfu L. Liang H. Wang G. Su X. Li L. Du Z.. (2016). miR-183 regulates autophagy and apoptosis in colorectal cancer through targeting of UVRAG. Oncotarget 7, 4735–4745. 10.18632/oncotarget.6732 26717041 PMC4826239

[B30] Jellinger K. A. (2003). Neuropathological spectrum of synucleinopathies. Mov. Disord. 18, S2–S12. 10.1002/mds.10557 14502650

[B31] Jellinger K. A. (2012). Neuropathology and pathophysiology of multiple system atrophy. Neuropathol. Appl. Neurobiol. 38, 379–380; author reply 381. 10.1111/j.1365-2990.2012.01268.x 22730560

[B32] Jellinger K. A. (2014). Neuropathology of multiple system atrophy: new thoughts about pathogenesis. Mov. Disord. 29, 1720–1741. 10.1002/mds.26052 25297524

[B33] Jing Z. Han W. Sui X. Xie J. Pan H. (2015). Interaction of autophagy with microRNAs and their potential therapeutic implications in human cancers. Cancer Lett. 356, 332–338. 10.1016/j.canlet.2014.09.039 25304373

[B34] Kabaria S. Choi D. C. Chaudhuri A. D. Mouradian M. M. Junn E. (2015). Inhibition of miR-34b and miR-34c enhances α-synuclein expression in Parkinson’s disease. FEBS Lett. 589, 319–325. 10.1016/j.febslet.2014.12.014 25541488 PMC4306645

[B35] Khoo S. K. Petillo D. Kang U. J. Resau J. H. Berryhill B. Linder J.. (2012). Plasma-based circulating MicroRNA biomarkers for Parkinson’s disease. J. Parkinsons Dis. 2, 321–331. 10.3233/JPD-012144 23938262

[B36] Kisos H. Pukaß K. Ben-Hur T. Richter-Landsberg C. Sharon R. (2012). Increased neuronal α-synuclein pathology associates with its accumulation in oligodendrocytes in mice modeling α-synucleinopathies. PLoS One 7:e46817. 10.1371/journal.pone.0046817 23077527 PMC3471961

[B37] Klionsky D. J. Abdelmohsen K. Abe A. Abedin M. J. Abeliovich H. Acevedo Arozena A.. (2016). Guidelines for the use and interpretation of assays for monitoring autophagy (3rd edition). Autophagy 12, 1–222. 10.1080/15548627.2015.1100356 26799652 PMC4835977

[B38] Klucken J. Poehler A. M. Ebrahimi-Fakhari D. Schneider J. Nuber S. Rockenstein E.. (2012). α-synuclein aggregation involves a bafilomycin A 1-sensitive autophagy pathway. Autophagy 8, 754–766. 10.4161/auto.19371 22647715 PMC3378419

[B39] Lang M. F. Yang S. Zhao C. Sun G. Murai K. Wu X.. (2012). Genome-wide profiling identified a set of miRNAs that are differentially expressed in glioblastoma stem cells and normal neural stem cells. PLoS One 7:e36248. 10.1371/journal.pone.0036248 22558405 PMC3340364

[B40] Lantos P. L. Papp M. I. (1994). Cellular pathology of multiple system atrophy: a review. J. Neurol. Neurosurg. Psychiatry 57, 129–133. 10.1136/jnnp.57.2.129 8126492 PMC1072436

[B41] Lee H. J. Cho E. D. Lee K. W. Kim J. H. Cho S. G. Lee S. J. (2013). Autophagic failure promotes the exocytosis and intercellular transfer of α-synuclein. Exp. Mol. Med. 45:e22. 10.1038/emm.2013.45 23661100 PMC3674407

[B43] Lee S. T. Chu K. Im W. S. Yoon H. J. Im J. Y. Park J. E.. (2011). Altered microRNA regulation in Huntington’s disease models. Exp. Neurol. 227, 172–179. 10.1016/j.expneurol.2010.10.012 21035445

[B42] Lee S. J. Desplats P. Sigurdson C. Tsigelny I. Masliah E. (2010). Cell-to-cell transmission of non-prion protein aggregates. Nat. Rev. Neurol. 6, 702–706. 10.1038/nrneurol.2010.145 21045796 PMC4996353

[B44] Lin H. Dai T. Xiong H. Zhao X. Chen X. Yu C.. (2010). Unregulated miR-96 induces cell proliferation in human breast cancer by downregulating transcriptional factor FOXO3a. PLoS One 5:e15797. 10.1371/journal.pone.0015797 21203424 PMC3009749

[B45] Lin S. Shao N. N. Fan L. Ma X. C. Pu F. F. Shao Z. W. (2014). Effect of microRNA-101 on proliferation and apoptosis of human osteosarcoma cells by targeting mTOR. J. Huazhong Univ. Sci. Technol. Med. Sci. 34, 889–895. 10.1007/s11596-014-1369-y 25480586

[B46] Liu N. Landreh M. Cao K. Abe M. Hendriks G. J. Kennerdell J. R.. (2012). The microRNA miR-34 modulates ageing and neurodegeneration in Drosophila. Nature 482, 519–523. 10.1038/nature10810 22343898 PMC3326599

[B47] Louis J. C. Magal E. Muir D. Manthorpe M. Varon S. (1992). CG-4, a new bipotential glial cell line from rat brain, is capable of differentiating *in vitro* into either mature oligodendrocytes or type-2 astrocytes. Front. Mol. Neurosci. 31, 193–204. 10.1002/jnr.490310125 1613821

[B48] Lynch-Day M. A. Mao K. Wang K. Zhao M. Klionsky D. J. (2012). The role of autophagy in Parkinson’s disease. Cold Spring Harb. Perspect. Med. 2:a009357. 10.1101/cshperspect.a009357 22474616 PMC3312403

[B49] Ma Y. Yang H.-Z. Dong B.-J. Zou H.-B. Zhou Y. Kong X.-M.. (2014). Biphasic regulation of autophagy by miR-96 in prostate cancer cells under hypoxia. Oncotarget 5, 9169–9182. 10.18632/oncotarget.2396 25333253 PMC4253426

[B50] Makioka K. Yamazaki T. Takatama M. Nakazato Y. Okamoto K. (2012). Activation and alteration of lysosomes in multiple system atrophy. Neuroreport 23, 270–276. 10.1097/WNR.0b013e3283503e4f 22343695

[B51] Masliah E. Rockenstein E. Mante M. Crews L. Spencer B. Adame A.. (2011). Passive immunization reduces behavioral and neuropathological deficits in an α-synuclein transgenic model of Lewy body disease. PLoS One 6:e19338. 10.1371/journal.pone.0019338 21559417 PMC3084838

[B52] Masuda-Suzukake M. Nonaka T. Hosokawa M. Kubo M. Shimozawa A. Akiyama H.. (2014). Pathological α-synuclein propagates through neural networks. Acta Neuropathol. Commun. 2:88. 10.1186/s40478-014-0088-8 25095794 PMC4147188

[B53] Matsuo A. Akiguchi I. Lee G. C. McGeer E. G. McGeer P. L. Kimura J. (1998). Myelin degeneration in multiple system atrophy detected by unique antibodies. Am. J. Pathol. 153, 735–744. 10.1016/s0002-9440(10)65617-9 9736024 PMC1853025

[B54] Mellios N. Huang H. S. Grigorenko A. Rogaev E. Akbarian S. (2008). A set of differentially expressed miRNAs, including miR-30a-5p, act as post-transcriptional inhibitors of BDNF in prefrontal cortex. Hum. Mol. Genet. 17, 3030–3042. 10.1093/hmg/ddn201 18632683 PMC2722882

[B55] Olanow C. W. Brundin P. (2013). Parkinson’s disease and α synuclein: is Parkinson’s disease a prion-like disorder? Mov. Disord. 28, 31–40. 10.1002/mds.25373 23390095

[B56] Peltier H. J. Latham G. J. (2008). Normalization of microRNA expression levels in quantitative RT-PCR assays: identification of suitable reference RNA targets in normal and cancerous human solid tissues. RNA 14, 844–852. 10.1261/rna.939908 18375788 PMC2327352

[B57] Pickford F. Masliah E. Britschgi M. Lucin K. Narasimhan R. Jaeger P. A.. (2008). The autophagy-related protein beclin 1 shows reduced expression in early Alzheimer disease and regulates amyloid β accumulation in mice. J. Clin. Invest. 118, 2190–2199. 10.1172/JCI33585 18497889 PMC2391284

[B58] Prusiner S. B. Woerman A. L. Mordes D. A. Watts J. C. Rampersaud R. Berry D. B.. (2015). Evidence for α-synuclein prions causing multiple system atrophy in humans with parkinsonism. Proc. Natl. Acad. Sci. U S A 112, E5308–E5317. 10.1073/pnas.1514475112 26324905 PMC4586853

[B59] Qiu L. Zhang W. Tan E. K. Zeng L. (2014). Deciphering the function and regulation of microRNAs in Alzheimer’s disease and Parkinson’s disease. ACS Chem. Neurosci. 5, 884–894. 10.1021/cn500149w 25210999

[B60] Reyes J. F. Rey N. L. Bousset L. Melki R. Brundin P. Angot E. (2014). α-synuclein transfers from neurons to oligodendrocytes. Glia 62, 387–398. 10.1002/glia.22611 24382629

[B61] Rickham P. P. (1964). Human experimentation. Code of ethics of the world medical association. Declaration of helsinki. Br. Med. J. 2:177. 10.1136/bmj.2.5402.177 14150898 PMC1816102

[B62] Sandri M. (2012). FOXOphagy path to inducing stress resistance and cell survival. Nat. Cell Biol. 14, 786–788. 10.1038/ncb2550 22854812

[B63] Schubert D. Heinemann S. Carlisle W. Tarikas H. Kimes B. Patrick J.. (1974). Clonal cell lines from the rat central nervous system. Nature 249, 224–227. 10.1038/249224a0 4151463

[B64] Schwarz L. Goldbaum O. Bergmann M. Probst-Cousin S. Richter-Landsberg C. (2012). Involvement of macroautophagy in multiple system atrophy and protein aggregate formation in oligodendrocytes. J. Mol. Neurosci. 47, 256–266. 10.1007/s12031-012-9733-5 22411133

[B65] Shults C. W. Rockenstein E. Crews L. Adame A. Mante M. Larrea G.. (2005). Neurological and neurodegenerative alterations in a transgenic mouse model expressing human α-synuclein under oligodendrocyte promoter: implications for multiple system atrophy. J Neurosci 25, 10689–10699. 10.1523/JNEUROSCI.3527-05.2005 16291942 PMC6725840

[B66] Spencer B. Desplats P. A. Overk C. R. Valera-Martin E. Rissman R. A. Wu C.. (2016). Reducing endogenous α-synuclein mitigates the degeneration of selective neuronal populations in an Alzheimer’s disease transgenic mouse model. J. Neurosci. 36, 7971–7984. 10.1523/JNEUROSCI.0775-16.2016 27466341 PMC4961781

[B67] Spencer B. Potkar R. Trejo M. Rockenstein E. Patrick C. Gindi R.. (2009). Beclin 1 gene transfer activates autophagy and ameliorates the neurodegenerative pathology in α-synuclein models of Parkinson’s and Lewy body diseases. J. Neurosci. 29, 13578–13588. 10.1523/JNEUROSCI.4390-09.2009 19864570 PMC2812014

[B68] Stefanova N. Reindl M. Neumann M. Kahle P. J. Poewe W. Wenning G. K. (2007). Microglial activation mediates neurodegeneration related to oligodendroglial α-synucleinopathy: implications for multiple system atrophy. Mov. Disord. 22, 2196–2203. 10.1002/mds.21671 17853477

[B69] Sun Y. Luo Z. M. Guo X. M. Su D. F. Liu X. (2015). An updated role of microRNA-124 in central nervous system disorders: a review. Front. Cell. Neurosci. 9:193. 10.3389/fncel.2015.00193 26041995 PMC4438253

[B70] Tan L. Yu J. T. Tan L. (2015). Causes and consequences of microRNA dysregulation in neurodegenerative diseases. Mol. Neurobiol. 51, 1249–1262. 10.1007/s12035-014-8803-9 24973986

[B71] Tan L. Yu J. T. Tan M. S. Liu Q. Y. Wang H. F. Zhang W.. (2014). Genome-wide serum microRNA expression profiling identifies serum biomarkers for Alzheimer’s disease. J. Alzheimers Dis. 40, 1017–1027. 10.3233/JAD-132144 24577456

[B72] Tiscornia G. Singer O. Verma I. M. (2006). Production and purification of lentiviral vectors. Nat. Protoc. 1, 241–245. 10.1038/nprot.2006.37 17406239

[B73] Tong J. Wong H. Guttman M. Ang L. C. Forno L. S. Shimadzu M.. (2010). Brain α-synuclein accumulation in multiple system atrophy, Parkinson’s disease and progressive supranuclear palsy: a comparative investigation. Brain 133, 172–188. 10.1093/brain/awp282 19903734

[B74] Ubhi K. Low P. Masliah E. (2011). Multiple system atrophy: a clinical and neuropathological perspective. Trends Neurosci. 34, 581–590. 10.1016/j.tins.2011.08.003 21962754 PMC3200496

[B75] Ubhi K. Rockenstein E. Doppler E. Mante M. Adame A. Patrick C.. (2009). Neurofibrillary and neurodegenerative pathology in APP-transgenic mice injected with AAV2-mutant TAU: neuroprotective effects of Cerebrolysin. Acta Neuropathol. 117, 699–712. 10.1007/s00401-009-0505-4 19252918 PMC3049872

[B76] Ubhi K. Rockenstein E. Kragh C. Inglis C. Spencer B. Michael S.. (2014). Widespread microRNA dysregulation in multiple system atrophy—disease-related alteration in miR-96. Eur. J. Neurosci. 39, 1026–1041. 10.1111/ejn.12444 24304186 PMC4052839

[B77] Valera E. Ubhi K. Mante M. Rockenstein E. Masliah E. (2014). Antidepressants reduce neuroinflammatory responses and astroglial α-synuclein accumulation in a transgenic mouse model of multiple system atrophy. Glia 62, 317–337. 10.1002/glia.22610 24310907 PMC4183229

[B78] Vallelunga A. Ragusa M. Di Mauro S. Iannitti T. Pilleri M. Biundo R.. (2014). Identification of circulating microRNAs for the differential diagnosis of Parkinson’s disease and Multiple System Atrophy. Front. Cell. Neurosci. 8:156. 10.3389/fncel.2014.00156 24959119 PMC4051126

[B79] Vilardo E. Barbato C. Ciotti M. Cogoni C. Ruberti F. (2010). MicroRNA-101 regulates amyloid precursor protein expression in hippocampal neurons. J. Biol. Chem. 285, 18344–18351. 10.1074/jbc.M110.112664 20395292 PMC2881760

[B80] Wenning G. K. Ben Shlomo Y. Magalhães M. Daniel S. E. Quinn N. P. (1994). Clinical features and natural history of multiple system atrophy. An analysis of 100 cases. Brain 117, 835–845. 10.1093/brain/117.4.835 7922469

[B81] Wenning G. K. Seppi K. Scherfler C. Stefanova N. Puschban Z. (2001). Multiple system atrophy. Semin. Neurol. 21, 33–40. 10.1055/s-2001-13117 11346023

[B82] Wenning G. K. Stefanova N. Jellinger K. A. Poewe W. Schlossmacher M. G. (2008). Multiple system atrophy: a primary oligodendrogliopathy. Ann. Neurol. 64, 239–346. 10.1002/ana.21465 18825660

[B83] Winslow A. R. Rubinsztein D. C. (2011). The Parkinson disease protein α-synuclein inhibits autophagy. Autophagy 7, 429–431. 10.4161/auto.7.4.14393 21157184 PMC3127221

[B84] Wong E. Cuervo A. M. (2010). Autophagy gone awry in neurodegenerative diseases. Nat. Neurosci. 13, 805–811. 10.1038/nn.2575 20581817 PMC4038747

[B85] Wu D. Jiang H. Chen S. Zhang H. (2015). Inhibition of microRNA-101 attenuates hypoxia/reoxygenationinduced apoptosis through induction of autophagy in H9c2 cardiomyocytes. Mol. Med. Rep. 11, 3988–3994. 10.3892/mmr.2015.3215 25606826

[B86] Xu Y. An Y. Wang Y. Zhang C. Zhang H. Huang C.. (2013). miR-101 inhibits autophagy and enhances cisplatin-induced apoptosis in hepatocellular carcinoma cells. Oncol. Rep. 29, 2019–2024. 10.3892/or.2013.2338 23483142

[B87] Yang J. Chen D. He Y. Meléndez A. Feng Z. Hong Q.. (2013). MiR-34 modulates Caenorhabditis elegans lifespan via repressing the autophagy gene atg9. Age (Dordr) 35, 11–22. 10.1007/s11357-011-9324-3 22081425 PMC3543738

[B88] Yu Y. Yang L. Zhao M. Zhu S. Kang R. Vernon P.. (2012). Targeting microRNA-30a-mediated autophagy enhances imatinib activity against human chronic myeloid leukemia cells. Leukemia 26, 1752–1760. 10.1038/leu.2012.65 22395361

[B89] Zhu H. Wu H. Liu X. Li B. Chen Y. Ren X.. (2009). Regulation of autophagy by a beclin 1-targeted microRNA, miR-30a, in cancer cells. Autophagy 5, 816–823. 10.4161/auto.9064 19535919 PMC3669137

