# Supplementary material for: MicroRNA-101 Modulates Autophagy and Oligodendroglial Alpha-Synuclein Accumulation in Multiple System Atrophy
Source: Front Mol Neurosci. 2017 Oct 17;10:329. doi: 10.3389/fnmol.2017.00329 (PMC5650998; doi:10.3389/fnmol.2017.00329)
Supplement: Supplementary file 1 [file Table_1.pdf]

GROUP	*N*	*AGE (years)*	*GENDER (F/M)*	*DURATION (years)*	*BRAAK*	*PMI (hours)*
Control	7	71.6 ± 5.9	4/3	N/A	0-1	15 ± 6
MSA-P	17	70.3 ± 9.8	10/7	7 ± 3	0-5	8 ± 7

Supplementary Table 1. Human brain tissue sample information. Information of human samples of healthy controls and MSA patients include age (years), gender, diagnosis, duration of the disease (years), Braak stage, and postmortem interval (PMI) (hours). Frozen, unfixed brain tissue samples were obtained from the University of California, San Diego Shiley-Marcos AD Research Center; Johns Hopkins Medical Institution Brain Resource Center; and Banner Sun Health Research Institute. MSA-P, MSA parkinsonian subtype. N/A, not applicable. Age, duration of the disease, and PMI hours are presented as average ± standard deviation.
