# Supplementary material for: MicroRNA-101 Modulates Autophagy and Oligodendroglial Alpha-Synuclein Accumulation in Multiple System Atrophy
Source: Front Mol Neurosci. 2017 Oct 17;10:329. doi: 10.3389/fnmol.2017.00329 (PMC5650998; doi:10.3389/fnmol.2017.00329)
Supplement: Supplementary file 3 [file Image_2.pdf]

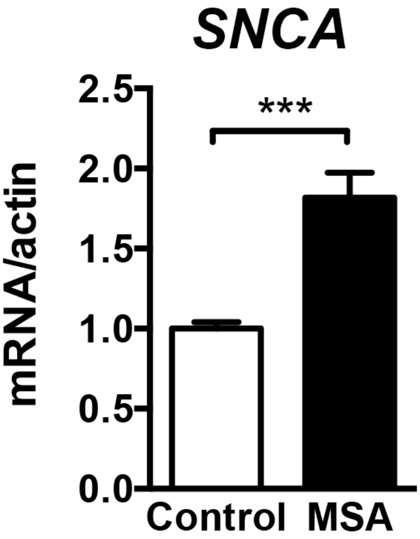

Supplementary Figure 2. Expression of SNCA in the striatum of MSA cases. qPCR analysis of the relative mRNA levels of human *SNCA* (α-syn gene) in the striatum of healthy controls and MSA patients. Results are expressed as averages +/- SEM. Statistical analysis was performed by Student's t-test. ***p<0.001.
