# Supplementary material for: MicroRNA-101 Modulates Autophagy and Oligodendroglial Alpha-Synuclein Accumulation in Multiple System Atrophy
Source: Front Mol Neurosci. 2017 Oct 17;10:329. doi: 10.3389/fnmol.2017.00329 (PMC5650998; doi:10.3389/fnmol.2017.00329)
Supplement: Supplementary file 4 [file Image_3.pdf]

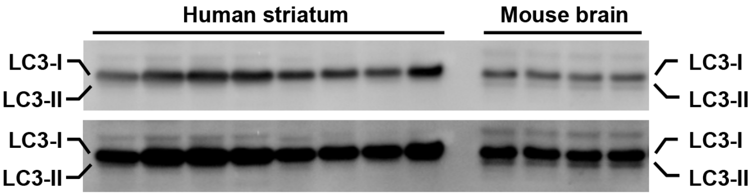

Supplementary Figure 3. Relative abundance of LC3-I and LC3-II in human vs. mouse brain homogenates. Representative results of LC3 immunostaining in protein homogenates from the striatum of human cases compared to protein homogenates obtained from mouse brains. Lower (top) and higher (bottom) levels of blot exposure are shown. A band corresponding to LC3-II is clearly observed exclusively in mouse brain homogenates.
