# Supplementary material for: MicroRNA-101 Modulates Autophagy and Oligodendroglial Alpha-Synuclein Accumulation in Multiple System Atrophy
Source: Front Mol Neurosci. 2017 Oct 17;10:329. doi: 10.3389/fnmol.2017.00329 (PMC5650998; doi:10.3389/fnmol.2017.00329)
Supplement: Supplementary file 5 [file Image_4.pdf]

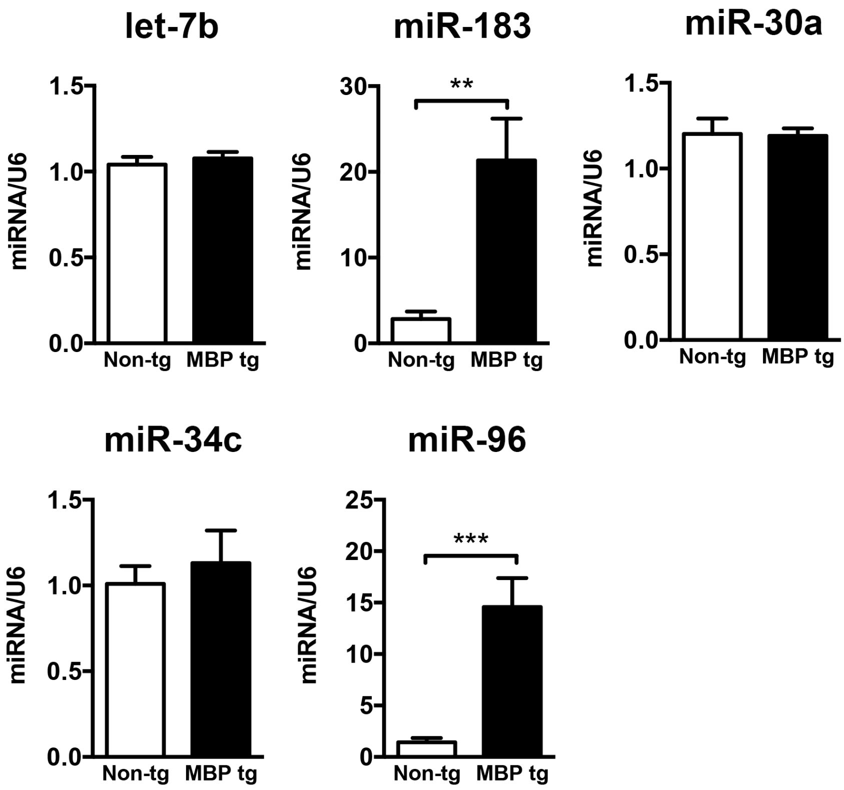

Supplementary Figure 4. Levels of miRNAs regulating the expression of autophagy proteins in the striatum of MBP-α-syn transgenic mice. qPCR analysis of the relative levels of let-7b, miR-183, miR-30a, miR-34c and miR-96 in the striatum of non-tg and MBP-α-syn tg mice. Results are expressed as averages +/- SEM. Statistical analysis was performed by Student's t-test. **$p<0.01$, ***$p<0.001$.
