# Supplementary material for: MicroRNA-101 Modulates Autophagy and Oligodendroglial Alpha-Synuclein Accumulation in Multiple System Atrophy
Source: Front Mol Neurosci. 2017 Oct 17;10:329. doi: 10.3389/fnmol.2017.00329 (PMC5650998; doi:10.3389/fnmol.2017.00329)
Supplement: Supplementary file 6 [file Image_5.pdf]

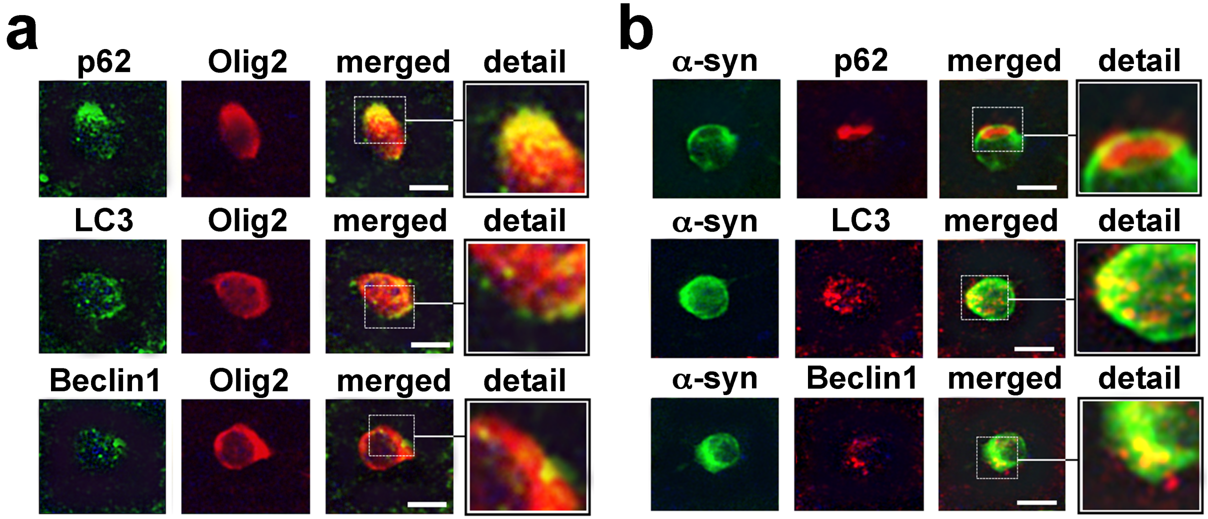

Supplementary Figure 5. Co-localization of autophagy proteins within oligodendrocytes and α-syn-positive cells in the striatum of MBP-α-syn tg mice. Selected microscopy images and magnified detail showing subcellular localization of the autophagy proteins p62, LC3 and Beclin 1 within (a) Olig2-positive cells and (b) α-syn-positive cells in the striatum of MBP-α-syn tg mice.
